# The Biology and Therapeutic Potential of the Src-YAP Axis in Non-Small Cell Lung Cancer (NSCLC)

**DOI:** 10.3390/cancers14246178

**Published:** 2022-12-14

**Authors:** Kostas A. Papavassiliou, Athanasios G. Papavassiliou

**Affiliations:** 1 First University Department of Respiratory Medicine, “Sotiria” Hospital, Medical School, National and Kapodistrian University of Athens, 11527 Athens, Greece; 2 Department of Biological Chemistry, Medical School, National and Kapodistrian University of Athens, 11527 Athens, Greece

## 1. Introduction

Non-small cell lung cancer (NSCLC) is the most common lung cancer type which accounts for the majority (~85%) of all lung cancer cases [1]. It is further classified into subtypes, including adenocarcinoma, squamous cell carcinoma, and large cell carcinoma. Due to technological advancements in molecular biology over the last decade, there have been significant improvements with regard to our understanding of the molecular pathobiology of these histological NSCLC subtypes. Research has translated this knowledge into major breakthroughs in the clinical diagnosis and management of NSCLC. NSCLC, particularly lung adenocarcinoma and squamous cell carcinoma, has been associated with oncogenic driver mutations. Most of these mutations occur in transmembrane receptors or intracellular protein kinases and result in the abnormal activation of signal transduction pathways which crosstalk with each other, leading to the uncontrolled growth, proliferation, and survival of NSCLC cells. Src is a non-receptor tyrosine kinase that plays an important role in the development and progression of NSCLC, acting downstream of multiple oncogenic growth factor receptors [2]. Likewise, the aberrant stimulation of the Hippo/Yes-associated protein (YAP) pathway has been associated with the promotion of cancer progression, metastasis, and drug resistance in NSCLC [3]. Substantial evidence suggests that Src kinases crosstalk with the Hippo/YAP signaling pathway, regulating the activation of YAP via several mechanisms. Additionally, data from studies focusing on this crosstalk demonstrate that the inhibition of the Src-YAP axis at multiple points represents a promising therapeutic strategy for the clinical management of advanced NSCLC. Thus, it is critical to understand the molecular details of how Src kinases interact with YAP in NSCLC cells.

### The Review by Hsu P.-C. et al., Published in Cancers

Hsu P.-C. and co-authors [4] provide an in-depth review of the role of the Src-YAP signaling axis in NSCLC. Initially, the authors present data on how YAP is potentiated by Src under physiological conditions in normal tissue, as well as in human diseases besides cancer. In order to induce the regeneration of intestinal mucosal epithelium after injury, glycoprotein 130 (gp130) triggers the activation of the tyrosine kinases Src and Yes, which, in turn phosphorylate and activate YAP to enter the nucleus and upregulate the expression of growth factor genes [5]. The crosstalk between Src and the Hippo/YAP pathway is also involved in the pathological setting of fibrosis and inflammatory disorders. Studies show that in fibroblasts, YAP accumulates in the nucleus under the control of actomyosin and Src family kinases. Furthermore, in a chronic kidney disease mouse model, it was demonstrated that the Src-YAP signaling axis promotes renal fibrosis [6,7,8,9]. Cancer-associated fibroblasts (CAFs), a group of activated fibroblasts with significant heterogeneity and plasticity in the tumor microenvironment, also depend on this crosstalk between YAP and Src kinases to activate themselves and maintain their tumor-promoting functions [10,11].

Next, Hsu P.-C. et al. discuss the different mechanisms through which the Src-YAP axis is upregulated. Within cancer cells, Src kinases activate YAP via the following three main mechanisms: (a) direct YAP phosphorylation, (b) the activation of pathways that lead to the repression of Hippo kinases, and (c) mechanisms that do not depend on the Hippo signaling pathway. YAP is mainly phosphorylated in a direct manner via Src kinases at tyrosine residue 357 to promote tumorigenesis in many cancers, including colorectal cancer, squamous cell carcinomas, and cholangiocarcinoma [12,13,14].

In an indirect manner, Src is able to activate YAP by repressing the Hippo-associated kinase large tumor suppressor homolog (LATS). A study in different cancer cell types revealed that Src disinhibits YAP by negatively regulating the G-protein-coupled receptor (GPCR) kinase 2 interacting protein-1 (GIT1), which promotes the LATS-mediated phosphorylation of YAP [15,16]. Another study found that the tissue inhibitor of metalloproteinase-1 (TIMP-1) activates Src, promoting the assembly of F-actin via RhoA, which leads to the repression of LATS and ultimately to YAP activation [17]. There is abundant evidence suggesting that the Src-mediated activation of downstream pathways, such as mitogen-activated protein kinase (MAPK) and phosphoinositide 3-kinase (PI3K) signaling pathways, results in the inhibition of Hippo kinases and, thus, the activation of YAP [18,19,20,21,22].

The last mechanism that is discussed by the authors of the review does not depend on the Hippo signaling cascade and does not involve direct YAP phosphorylation. One study demonstrated that integrin α3 acts via the focal adhesion kinase (FAK)/Src-cell division control protein 42 homolog (CDC42)–protein phosphatase 1 α (PP1A) axis to regulate the phosphorylation and transcriptional activation of YAP in transit-amplifying cells and that this activation leads to the upregulation of mechanistic target of rapamycin (mTOR) signaling [23].

To further emphasize the significance of the Src-YAP signaling axis in the pathobiology of NSCLC, the authors of the review discuss its association with the development of resistance to targeted therapy. Using transposon mutagenesis and clinical genomics, Fan et al. demonstrated that the amplification of YAP1 is a mechanism of acquired resistance to epidermal growth factor receptor (EGFR) inhibitors in EGFR-mutant NSCLC patients [24]. Chaib et al. showed that the co-activation of signal transducer and activator of transcription 3 (STAT3) and Src-YAP signaling in EGFR-mutant NSCLC functions to promote intrinsic and acquired resistance to EGFR tyrosine kinase inhibitors (TKIs). Further in vitro and in vivo data revealed that the combination of an EGFR along with an Src inhibitor had a synergistic antitumor effect [20]. In another study using *K-ras* mutant NSCLC cells, the combination of an MAPK kinase (MEK) inhibitor with an Src kinase inhibitor downregulated the expression of YAP and resulted in tumor growth suppression in NSCLC xenograft mouse models [25]. Such studies indicate that resistance to TKIs for NSCLC may be overcome by co-targeting the Src-YAP signaling axis. Therefore, research into the development of inhibitors that specifically target the Src-YAP axis at different points is warranted.

Finally, Hsu P.-C. et al. elaborate on potential therapies that target the Src-YAP signaling axis and include an informative table which lists all of these therapies. The first category of therapeutics includes the three Src inhibitors, namely, dasatinib, bosutinib, and saracatinib, all of which are under investigation for the treatment of NSCLC patients in clinical trials [26,27,28]. The second category includes small-molecule inhibitors of intracellular signaling pathways acting downstream of Src and leading to YAP activation, such as MAPK, PI3K, Rho/Rho-associated protein kinase (ROCK), and STAT3 signaling [20,29,30,31]. The third category of Src-YAP targeting agents includes inhibitors that act to disrupt the YAP-transcriptional enhanced associate domain (TEAD) complex, thus blocking the transcriptional activity of YAP [32,33,34,35]. Lastly, the fourth category includes cyclin-dependent kinase (CDK) inhibitors (CDKis). CDKs are protein kinases that regulate the cell cycle, and there is evidence that some CDKs can phosphorylate YAP and, hence, affect the transcription of YAP target genes [36,37,38,39]. Some CDKis under evaluation for the treatment of NSCLC are the CDK1,5,9 inhibitor dinaciclib [40,41], the CDK7 inhibitor THZ1 [37,38], and the CDK9 inhibitors seliciclib [40] and flavopiridol [39,40].

To summarize, Hsu P.-C. et al. present a comprehensive review on the role of the Src-YAP signaling axis in the initiation and progression, as well as the drug resistance, of NSCLC and emphasize the therapeutic potential of targeting this oncogenic crosstalk with various agents. From their work, it becomes apparent that the interaction of Src with the Hippo/YAP signaling pathway is complex, and further research is required in order to dissect its molecular underpinnings. From a clinical perspective, the Src-YAP axis is a promising therapeutic target, and given its complexity, it may be targeted at multiple points. Notably, YAP has been identified as a mechanotransducer in normal and cancer cells, transducing the mechanical cues that are sensed outside of the cell at the molecular level to modulate gene expression [42,43,44]. Src kinases are also associated with the mechanobiological aspects of cancer cells [45]. As mechanosignaling plays a key role in the promotion of the malignant features of lung cancer cells [46,47], it may be interesting to probe for potential mechanobiology-related crosstalk between Src and YAP in NSCLC.

Despite the development of new therapeutics, including immunotherapies and targeted therapies, lung cancer remains the leading cause of cancer-related mortality worldwide. Based on this review by Hsu P.-C. et al. and other reports, it is likely that future research regarding the crosstalk between the Src and Hippo/YAP signaling pathways will reveal its biological significance in NSCLC and will serve for the discovery and development of novel therapeutic approaches which may enhance the clinical management of NSCLC patients.
